# Evidence to support a food-based dietary guideline on sugar consumption in South Africa

**DOI:** 10.1186/1471-2458-12-502

**Published:** 2012-07-04

**Authors:** Nelia P Steyn, Norman J Temple

**Affiliations:** 1 Centre for the Study of Social and Environmental Determinants of Nutrition; Population Health, Health Systems, and Innovation. Human Sciences Research Council, P Bag X9182, Cape Town, 8000, South Africa; 2 Centre for Science, Athabasca University, Athabasca, Alberta, Canada

## Abstract

**Background:**

To review studies undertaken in South Africa (SA) which included sugar intake associated with dental caries, non-communicable diseases, diabetes, obesity and/or micronutrient dilution, since the food-based dietary guideline: *“Use foods and drinks that contain sugar sparingly and not between meals”* was promulgated by the Department of Health (DOH) in 2002.

**Methods:**

Three databases (PubMed, Cochrane Library, and ScienceDirect), and SA Journal of Clinical Nutrition (SAJCN), DOH and SA Medical Research Council (SAMRC) websites were searched for SA studies on sugar intake published between 2000 and January 2012. Studies were included in the review if they evaluated the following: sugar intake and dental caries; sugar intake and non-communicable diseases; sugar and diabetes; sugar and obesity and/or sugar and micronutrient dilution.

**Results:**

The initial search led to 12 articles in PubMed, 0 in Cochrane, 35 in ScienceDirect, 5 in the SAJCN and 3 reports from DOH/SAMRC. However, after reading the abstracts only 7 articles from PubMed, 4 from SAJCN and 3 reports were retained for use as being relevant to the current review. Hand searching of reference lists of SAJCN articles produced two more articles. Intake of sugar appears to be increasing steadily across the South African (SA) population. Children typically consume about 50 g per day, rising to as much as 100 g per day in adolescents. This represents about 10% of dietary energy, possibly as much as 20%. It has been firmly established that sugar plays a major role in development of dental caries. Furthermore, a few studies have shown that sugar has a diluting effect on the micronutrient content of the diet which lowers the intake of micronutrients. Data from numerous systematic reviews have shown that dietary sugar increases the risk for development of both obesity and type 2 diabetes. Risk for development of these conditions appears to be especially strong when sugar is consumed as sugar-sweetened beverages.

**Conclusion:**

Based on the evidence provided the current DOH food-based dietary guideline on sugar intake should remain as is.

## Background

In 2002 the Department of Health in South Africa (SA) adopted food-based dietary guidelines, including one on sugar stating: “Use food and drinks containing sugar sparingly and not between meals” [1]. Food-based dietary guidelines are simple dietary messages aimed at improving the health of the population by focusing on relevant nutritional issues. South Africa currently has eleven such guidelines which are a public health measure for preventing nutritional disorders. These are shown in Table 1.

**Table 1:** South African food-based dietary guidelines approved by Department of Health as a health promotion tool

**Food-based dietary guideline**	**Underlying reason for guideline**
Enjoy a variety of foods	Protection against micronutrient deficiencies
Be active	Prevention of non-communicable diseases
Make starchy foods the basis of most meals	Emphasising that carbohydrates also play a role. Also prevention of under-nutrition
Eat dry beans, peas, lentils and soya regularly	Affordable source of protein and minerals
Chicken, fish, milk, meat or eggs can be eaten daily	Promoting sources of animal protein and contributing to iron and calcium needs
Drink lots of clean safe water	To maintain healthy body hydration
Eat plenty of fruit and vegetables every day	For prevention of non-communicable diseases and micronutrient deficiencies. A
Eat fats sparingly	Prevention of non-communicable diseases
Use salt sparingly	Prevention of hypertension
Use food and drinks containing sugar sparingly and not between meals	Prevention of dental caries
If you drink alcohol, drink sensibly	Prevention of chronic alcoholism and micronutrient deficiencies

The food-based guideline on sugar intake was based on evidence published by the WHO/FAO Expert Consultation Group [2] as well as evidence from local SA studies published in peer reviewed journals up to, and including, the time the manuscript was published. The authors believed that there was sufficient evidence available at the time showing an association between a high sugar intake and increased risk of dental caries and obesity. At the time increased risk due to sugar intake was not associated with any other disease conditions.

## Methods

In the current paper the authors reviewed new evidence published from 2000 up to January 2012, in order to re-evaluate the appropriateness of the “sugar” guideline. There were two facts which hampered this assessment: first, there were no national dietary data for adult South Africans, and, secondly, the most recent national dental data were from 1999–2002 [3,4].

In order to find recent data on sugar intake in South Africa a review was undertaken and the following databases were searched for original research studies (descriptive or other) in SA since 2000 using the search terms: *sugar AND dental caries AND South Africa; sugar AND non-communicable diseases AND South Africa; sugar AND obesity AND South Africa; sugar AND diabetes AND South Africa; sugar AND micronutrient dilution AND South Africa.* PubMed, Cochrane Library, and ScienceDirect were searched as well as the website of the local nutrition journal- South African Journal of Clinical Nutrition (SAJCN). Additionally, the websites of the SA Medical Research Council (SAMRC) and Department of Health were searched for data on national surveys. In this publication, sugar or sucrose, refers to “added sugar “which refers to all mono-and disaccharides added to foods and drinks in preparation and cooking.

## Results and discussion

The initial search led to 12 articles in PubMed, 0 in Cochrane, 35 in ScienceDirect, 5 in the SAJCN and 3 reports from Department of Health/SAMRC (Table 2). However, after reading the abstracts only 7 articles from PubMed [1,5-10], 4 from SAJCN [11-14] and 3 reports [4,15,16] were retained for use in the manuscript as being relevant to the current review. Hand searching of reference lists of SAJCN articles produced two more articles [17,18].

**Table 2:** Relevant articles generated by using the search terms “sugar” AND “South Africa” AND Terms provided below

**Sugar AND South Africa AND**	**PubMed**	**Cochrane**	**ScienceDirect**	**SAJCN**	**Reports**
Dental caries	7	0	9	0	2(2)
Non-communicable diseases	0	0	1	0	0
Diabetes	3	0	14	0	0
Obesity	1	0	11	0	0
Micronutrient dilution OR dietary intake	1	0	0	5	1
Total articles retrieved	12	0	35	5	3
Articles of relevant studies after studying abstracts	7	0	0	5	3

### Sugar consumption in South Africa

The only national data on sugar consumption in SA comes from the 1999 National Food Consumption Survey (NFCS) in 1 to 9 year old children [15]. The most commonly consumed sources of added sugar in the diet came from table sugar, sweetened squash (sweetened concentrate to which water is added), jam, cookies, sweetened soft drinks (carbonated), sweets (candy) and breakfast cereals (in decreasing order of frequency).

Sugar intake in 6–9 year olds in SA ranged from 22 g in the Eastern Cape to 57 g in the Western Cape Province [16]. This amounted to a mean intake of 42 g in urban areas and 26 g in rural areas; 67 g in white children and 47 g in black children. In urban areas sugar intake exceeded the 10 %E recommended by the WHO [2]. Further analyses of the NFCS data revealed that in 1–9 year olds sugar contributed 5.5 % to overall energy intake and 9 % to carbohydrate intake [14]. The average portion size of sugar was 21.3 g per day. Squash (300 g) contributed an average of 10.4 g and carbonated drinks (300 g) an average of 6 g sugar per day.

Three studies on dietary intake in school children were undertaken in the Vaal region , Pietermaritzburg and in Cape Town. Oosthuizen et al. [13] reported that the majority of children consumed numerous items high in sugar, including cookies (mean = 59 g), sweets (mean = 41 g) and squash (mean = 316 g). The study on 11 schools in Pietermaritzburg showed that children obtained 34 g added sugar from soft drinks at school, 13 g from flavoured milk, and 24 g from mixed fruit blends [12]. Packets of sweets and lollipops were available at most school shops and provided 69.1 g and 29.4 g sugar respectively per serving. Similarly, in 14 Cape Town schools 70 % of children purchased no healthy items while 73.2 % purchased two or more unhealthy (high in fat/sugar) items at the school shops [8].

Lourens et al. [17] assessed soft drink consumption of children in Wynberg, Cape Town (Table 3). Children in grades 4 and 7 at two government schools in this low-to-medium socio-economic suburb were interviewed. The average consumption of soft drinks was found to be 730 g (SD 530) per child per day (i.e. about 2 servings at 350 g each). This implies that these children have a sugar intake of 40–80 g per day from soft drinks alone. Unfortunately, intake from sweetened fruit juices (fruit juice with sugar added) were not assessed.

**Table 3:** Consumption of sweetened soft drinks by children at a school in Wynlands, Cape Town

	**All**	**Grade 4**	**Grade 7**	**All boys**	**All girls**
Mean amount ml/g/day	730	725	734	819	655
SD	530	500	556	552	501

Source: Adapted from Louwrens, Venter & Otty, 2010 [17].

A study of adolescents in Gauteng revealed alarming trends in intake of added sugar [18]. At age 10 years (year 2000) their mean intake of sugar was 68 g (CI 62.5-73.1) per day. This represented 16 % of energy (E) intake. At age 13 years (year 2003) intake for the same adolescents had risen to 102 g (CI 95.0-109.7) per day (20 % E); this is a very high intake. It is possible, however, that these figures may have been overestimated since it is known that the food frequency method does tend to overestimate dietary intakes [19].

A secondary data analysis of numerous dietary studies in South Africa was undertaken in 2002 in order to extrapolate adult dietary intakes [16]. This showed that sweetened squash were commonly consumed by all age groups. Twenty-four percent of 6-9-year olds had consumed a sweetened beverage on the day prior to the study. Average amounts ranged from 326 g to 444 g per day.

With the exception of the **C**ardiovascular Risk Study **i**n Black South Africans (CRIBSA) there are few representative dietary studies in adults [20]. This study investigated dietary intake by 24-hour recall in a sample of urban adult (18–60 years) black South Africans residing in four townships in Cape Town, (n = 1010). The values for females ranged from 51 g (SD 40 g) in the youngest group to 38 g (SD 24 g) in the oldest and in males from 52 g (SD 49 g) to 51 g(SD 39 g) [9]. Sugar intake contributed 10.7 – 11.4 % of energy intake (E) in males and 14.6 -11.4 % E in women, values greater than the 10%E recommended by WHO [2].

In Table 4 food balance sheets are given for SA, USA, UK and a few neighbouring African countries. It is interesting to note that SA has a per capita (available) sugar intake similar to that of the UK and considerably higher than those of other African countries although it is still considerably less than that of the USA. Sugar-sweetened beverages (SSBs) are the top source of added sugar in the American diet [21]. Currently, added sugar intake of Americans is estimated to be about 15.8% of energy intake and sugars in soft drinks account for 47% of added sugars in the diet. SSBs contribute 8-9 % of energy [22].

**Table 4:** Per capita sugar intake of South Africa from food balance sheets

**Country**	**Sugar kg/per capita/year**	**Per capita gram per day**
USA	67.6	185
UK	36.2	99.2
South Africa	30.7	84.1
Kenya	18.3	50.1
Tanzania	10.4	28.5
Uganda	9.4	25.7
Malawi	8.8	24.1

Source: FAO Food Balance Sheets, 2007.

### Sugar and dental caries

Numerous studies over the past 50 years have confirmed the relationship between dietary sugars and dental caries. A classic example is the study of Japanese children after the Second World War whose DMFT (decayed, missing and filled teeth) values escalated from 2.8 in 1957 to 5.9 in 1975 [23]. This jump was associated with an increase in sugar consumption from around 35 g per capita per day in 1957 to 68 g in 1975. Sreebny analysed data from FAO food balance sheets of 47 nations and found that for 12-year-old children there was a significant positive correlation between per capita sugar available and dental caries [24]. More recently, a study in Australia carried out on primary school children reported that the development of caries was significantly associated with consumption of sweet drinks and sweet treats as well as suboptimal fluoride exposure and irregular dental care [25].

In simple terms, caries develops as a result of the metabolism of sugars and other fermentable carbohydrates to acids by plaque bacteria [26]. A low pH favours the growth of acidogenic bacteria such as streptococci, resulting in demineralization when the pH drops to <5.5, the critical value. The presence of fluoride, calcium and phosphates can raise the pH by about 0.5 pH units and thus have a preventive effect. Sucrose is regarded as being the most cariogenic sugar because of its ability to form glycan which increases adhesion to teeth, and because frequency of occurrence of sugar intake increases risk of caries as does sucrose in a form that is retained in the mouth for long periods [27].

Data from the most recent national Children’s Oral Health Survey (n = 5822) in South Africa (1999/2002) showed some interesting dental trends, namely that 39.7% of 6-year olds were caries free [3,4]; this is less than the DOH target of 50%. In older children (12-year olds) mean DMFT had decreased from 2.5 in 1982 to 1.1 in the 1999/2002 study. The authors ascribed this to the widespread use of fluoridated toothpaste which has been used over the past 20–30 years. Another important finding was that more than 80% of caries in preschool children goes untreated with caries being more severe in the primary than in the permanent dentition. Table 5 shows that the decayed component of the DMFT is high in all age groups while negligible levels are filled. This clearly shows that there are inadequate dental resources, dental facilities and lack of awareness about oral health in South Africa. Table 6 presents data on dental care. A large percentage of children and adults were found to need care (45-60 %). The mean number of teeth needing care per child ranged from 2 to 3.5. This emphasises the importance of health promotion regarding oral health and the prevention of dental caries, particularly in children. Postma et al. conducted further analyses on the data from the national study and found that increased per capita sugar expenditure and decreasing water fluoride levels were significantly associated with an increased risk for early childhood caries [5].

**Table 5:** The percentage prevalence of dental caries and untreated caries and the severity of caries by age group in South Africa

**Age**	**Weighted national mean**		**DMFT/dmft**	**D/d**	**M/m**	**F/f**
	**% caries**	**% untreated caries**				
4-5	50.6	46.6	2.4	2.0	0.4	0.2
6	60.3	55.1	2.9	2.2	0.5	0.1
12	36.9	30.3	1.1	0.8	0.2	0.1
15	51.0	42.2	1.9	1.3	0.3	0.2
35-44	95.0	68.0	12.3	3.2	8.2	1.2

Source: adapted from van Wyk and van Wyk, 2004 [3].

D = decayed, m = missing, f = filled; t = teeth.

**Table 6:** Percentage distribution of care needed, the mean number of teeth needing care and the type of care needed for the treatment of dental caries per age group in South Africa expressed as the mean number of teeth needing care

**Age groups**	**% Needing care**	**Mean number of teeth**	**Preventive**	**Restorations**	**Crown + Veneer**	**Pulp care**	**Extractions**
4-5*	45.6	2.1	0.5	0.9	0	0	0.6
6	59.1	3.0	1.0	1.0	0	0	0.9
12	45.3	2.6	1.8	0.6	0	0	0.2
15	49.9	2.9	1.5	1.0	0	0	0.2
35-44**		3.5					

Source: adapted from van Wyk & van Wyk, 2004* [3];

*Primary teeth; **1988/89.

Apart from the national oral health survey two local SA studies are also worth noting, since they attempted to define the relationship between caries and sugar intake in children. Researchers found a significant increase in prevalence of caries in children 1–5 years as energy, carbohydrate and added sugar increased in their sample of urban black children in Johannesburg (n = 1639) [9]. In older children (10–15 years) examined in Limpopo it was found that lower added sugar and higher fluoride intakes among rural Mahonisi children contributed to a lower caries experience [6]. MacKeown and Faber [7] showed that the rural/urban environment played a strong role on specific cariogenic food habits among young black SA children, with women in rural areas breastfeeding more frequently than urban ones and urban mothers were also more likely to add sugar to their infants’ pacifiers.

The question arising is how much sugar is permissible before it becomes a risk for development of dental caries? Some researchers have found a dose–response relationship between sugar intake and caries. This relationship is affected by the presence of fluoride. Sheiham [27], proposed an upper limit of 15 kg per person per year in the presence of adequate fluoride and 10 kg in its absence. This translates to an intake of 41 g and 27 g per day, respectively. Sreebny [24] suggested that 50 g per capita per day should be regarded as the upper limit of safe or acceptable sugar consumption after examining the food balance sheets and caries prevalence of nearly 50 countries.

Tinanhoff and Palmer [28] developed evidence-based dietary recommendations for reducing caries in preschool children. These include: avoid frequent consumption of juice or sugar-containing beverages; don’t allow infants to sleep with feeding bottles; avoid cariogenic snacks and limit cariogenic foods to mealtimes; restrict sugar-containing snacks that remain in the mouth for long periods or are eaten frequently (e.g. candy and lollipops); and, overall, have a balanced diet in accordance with the food guide. Further recommendations include appropriate use of fluoride, good oral hygiene and regular preventive and restorative dental care. Stephens et al. [29] stressed the importance of exclusive breastfeeding in the first 6 months of life in order to prevent caries.

### Sugar and malnutrition

Malnutrition in children is frequently an outcome of inappropriate child-feeding practices. This is commonly due to limited access to fresh nutrient-dense foods resulting in substitution of low-cost, energy-dense, nutrient-poor sugary and fatty foods [30]. This changed food environment may result from changes in traditional ethnic eating patterns or from food insecurity arising from poverty.

Sugar provides only empty calories and for that reason it has often been suggested that a high consumption of sugar displaces nutrients from the diet [31]. In two South African studies this has been documented [1,32]. Data from the NFCS was divided into tertiles according to sugar consumption. Protein intake was significantly lower in the highest tertile of sugar intake than in the lowest (40 vs 45 g per day; p = 0.04), as was protein as % E (12 vs 14 % E; p = 0.02) [1]. Thiamine intake was also significantly lower in the highest sugar tertile group compared with the lowest group (0.51 vs 0.65 mg per day; p = 0.0001). In a study of black elderly women energy, protein, fibre, vitamin B6, thiamine, folate, vitamin C, iron calcium, zinc and selenium intake were significantly lowest in women in the highest percentage energy tertile [32].

While a recent review on micronutrient dilution was inconclusive and cited methodological issues as being a considerable constraint to obtaining reliable evidence [33], two national studies have provided valuable data regarding nutrient dilution [34,35]. Joyce and Gibney [34] evaluated the Irish National Children’s Food Survey and the National Teen Food Survey data which used 7-day food diaries. Their results indicate that a high intake of added sugar is associated with decreased micronutrient density of the diet resulting in dietary inadequacies. The nutrients significantly affected were magnesium, calcium, zinc and vitamins B12 and C. Gibson and Boyd [35] evaluated the micronutrient density of the diets of British children, aged 4 to 18 years. The data came from the National Diet and Nutrition Survey which also used 7-day food diaries. They compared micronutrient intakes in low and high consumers of sugar (quintiles 1 and 5; 9 % and 23 % of E, respectively). Nutrients most affected negatively by a high sugar intake were zinc, magnesium, iron and vitamin A. Although the differences were relatively modest, it needs to be taken into consideration that intakes of most nutrients (except zinc) exceeded the reference intakes. The researchers suggested that the impact may be more severe when children are consuming inadequate amounts of nutrients.

### Sugar and obesity

The literature review on SA studies did not illicit any publications regarding a direct relationship between sugar intake and obesity; however the high prevalence of overweight and obesity in SA adolescents and adults is acknowledged [36,37]; as is the high intake of squash and soft drinks in children [17]. For example, 27.8 % of 13–19 year old females in SA were found to be overweight and obese (BMI > =25) [36] and this increased to 54.9 % % in adult women [37]. As mentioned earlier, the average consumption of soft drinks was found to be 730 g (SD 530) per child per day (i.e. about 2 servings at 350 g each) at schools in Cape Town [17].

In the USA and Europe much attention has been paid to the association between consumption of sugar-sweetened beverages (SSBs) and weight gain. On average 1 can of carbonated SSB (soft drink) provides 40–50 g of sugar and 150 calories, generally in the form of high-fructose corn syrup [38].

A systematic review by Malik et al. [39] which included 30 studies showed a positive association between intake of SSBs and weight gain in both children and adults. They concluded that “sufficient evidence exists for public health strategies to discourage consumption of sugary drinks as part of a healthy lifestyle.”

Strong supporting evidence for a role of SSBs in weight gain came from recent findings from two large prospective investigations carried out in the USA on 121 000 men and women over a period of 20 years [40]. The pooled results found that within each 4-year period subjects gained an average of 3.35 lb (CI −4.1-12.4). Intake of SSBs was one of the foods most strongly associated with this 4-year weight gain (1.00 lb). Associations for other foods were as follows: French fries (3.35 lb), potatoes (1.28 lb), red meat (0.95 lb), processed meat (0.93 lb), vegetables (−0.22 lb), whole grains (−0.37 lbs), fruits (−0.49 lbs), nuts (−0.57 lbs), and yoghurt (−0.82 lb)(p < =0.005 for each comparison).

The dramatic increase in obesity in the USA over the past few decades has been of much concern, especially as the condition is a risk factor for type 2 diabetes, cardiovascular diseases, and some types of cancer. Obesity is believed to be responsible for 9.1 % of the total health-care budget in the USA [41]. As sugar is related to obesity, reducing the intake of sugar is therefore a means to help prevent the other conditions. Based on current evidence the American Heart Association recently recommended that added sugar in the diet of Americans should not exceed 100–150 calories (25–37.5 g sugar) per day [42].

### Sugar and type 2 diabetes

The literature review on SA studies did not illicit any publications regarding a direct relationship between sugar intake and diabetes. However international reviews in this regard provide evidence of a strong association between SSBs and type 2 diabetes. Schulze et al. [43] examined the association between sugar sweetened drinks, weight gain and increased risk of type 2 diabetes (T2D) in a cohort of 91 249 women from the Nurses Study. They found that women who had 1 or more SSB per day over a 4 year period gained more weight than those who decreased their intake of SSBs after adjusting for lifestyle and other confounders. Furthermore women who had 1 or more SSBs had a relative risk (RR) of type 2 diabetes of 1.83 (CI 1.42-2.36; p < 001) compared with those who consumed less than 1 drink per day. Consumption of sweetened fruit juice conferred a 2.00 RR (CI = 1.33-3.03; p = 0.001) for having 1 or more drinks a day compared with having less than 1.

A meta-analyses recently published evaluated 11 studies with metabolic syndrome as outcomes of T2D [44]. Inclusion criteria were studies with a prospective cohort design, end points which included the metabolic syndrome (MS) or T2D, relative risk calculations and adjustment for confounders. This resulted in 11 studies on the MS and 8 on T2D. The studies included comprised 310 819 participants in total. Participants having 1–2 servings SSBs per day had a 1.26 (CI 1.12-1.41) greater risk of developing T2D and a 1.20 greater risk (CI = 1.02-1.42) of MS compared with those having less than 1 serving a month.

A study by De Koning et al. [45] included 40 389 healthy men from the Health Professionals Follow-up Study, a prospective study over 20 years. An association was tested between intake of SSBs and T2D. After 20 years 2680 cases of T2D were found. The hazard risk (HR) for SSB intake and T2D was 1.09 (p < 0.01) after adjusting for confounders. The HR was not significant when tested for artificially sweetened beverages.

### Cardiovascular disease risk

Although there is less evidence in this regard there is still sufficient to show that SSBs also impact on this area of health. Numerous large cohort studies have shown the effects of SSBs on blood pressure including the Framingham study and Nurses’ Health Study [46,47]. Framingham offspring (n = 6154) who consumed ≥ 1 SSB per day over 4 years had a 22% higher risk of hypertension than those who did not (RR = 1.22 (95% CI 1.05, 1.41)(47). In the Nurses’ Health Study 1 and 11, women who consumed ≥4 SSBs a day had a 44 % and a 22 % higher risk of hypertension, respectively (RR = 1.44; and RR = 1.28) [46].

Risk of developing coronary heart disease (CHD) was also studied in 88,000 women of the Nurses’ Health Study over 24 years [48]. Those who consumed ≥ 2 SSB per day had a 35 % greater risk of developing CHD compared with those consuming SSBs less than once a month (RR = 1.35(95 % CI 1.1, 1.7).

Blood lipids may also be detrimentally influenced by consumption of SSBs. The Framingham Offspring Study reported that those who consumed ≥1 SSB per day had a higher risk of developing both hypertriglyceridaemia (RR = 1.25 [CI 1.04-1.51]; n = 3800) and low HDL-cholesterol (RR = 1.32 [CI 1.06-1.64]; n = 3500) over 4 years compared to non-consumers [46].

#### What should health professionals recommend with regard to sugar intake in children and in adults?

A summary of the literature presented in this paper highlights the following current knowledge regarding the relationship between sugar intake and health and includes SA studies published since 2000: Firstly, sugar is clearly a major factor that causes caries. Secondly, a high intake of sugar inevitably leads to a reduced dietary intake of micronutrients. Thirdly, strong evidence has emerged in recent years that links intake of sugar, especially SSBs, with an increased risk of obesity and type 2 diabetes (T2D). This relationship may also be true for CHD.

The American Heart Association recently recommended that added sugar in the diet should not exceed 100 calories (25 g) per day for women or 150 calories (37.5 g) for men [30]. This is equivalent to about 5-6 % of dietary energy (E). A sugar intake of 10 % E should be seen as an acceptable upper limit. An intake of <6 % E is closer to the ideal intake and would apply especially to people at increased risk of the negative health consequences of sugar, such as those who are overweight, have pre-diabetes, or live in areas where the drinking water is not fluoridated. One tin of SSB (one serving) contains 350 ml and approximately 40 g of sugar; this is 150 calories or about 6-7 % of one day’s energy intake. It follows, therefore, that neither adults nor children should consume more than one serving of SSB per day.

In order to prevent frequent exposure to sugar a bottle-fed infant should not be given any beverage with added sugar or allowed to lie down with a bottle. Young children should not be given beverages with added sugar. Frequency of intake of sweetened foods/drinks should be reduced as much as possible, particularly in children, in order to help prevent dental caries.

## Conclusions

The current sugar guideline used in South Africa: ** *Use foods and drinks that contain sugar sparingly and not between meals* ** should remain as is, however the above points should be included in recommended dietary guidelines which accompany the food-based dietary guidelines.

## Competing interests

The authors declare that they have no competing interests.

## Authors' contributions

NS and NT jointly did a literature search and drafted the manuscript. Both authors read and approved the final manuscript.

## Pre-publication history

The pre-publication history for this paper can be accessed here:

http://www.biomedcentral.com/1471-2458/12/502/prepub
